# Caution for living donor liver transplantation with congenital portosystemic shunt: a case report

**DOI:** 10.1186/s40792-022-01533-3

**Published:** 2022-10-06

**Authors:** Yoshihiro Nagao, Katsuya Toshida, Akinari Morinaga, Takahiro Tomiyama, Yukiko Kosai, Tomonari Shimagaki, Takahiro Tomino, Huanlin Wang, Takeshi Kurihara, Takeo Toshima, Kazutoyo Morita, Shinji Itoh, Noboru Harada, Tomoharu Yoshizumi

**Affiliations:** 1 Department of Advanced Medical Initiatives, Kyushu University Hospital, Fukuoka, Japan; 2 Department of Surgery and Science, Graduate School of Medical Sciences, Kyushu University, 3-1-1 Maidashi, Higashi-Ku, Fukuoka, 812-8582 Japan

**Keywords:** Balloon-occluded retrograde transvenous obliteration, Donor hepatectomy, Hyperammonemia, Portal blood flow, Portosystemic shunt

## Abstract

**Background:**

Congenital portosystemic shunt is an infrequent abnormal connection between the portal vascular system and the systemic circulation. Portosystemic shunts are common findings in patients with cirrhosis, causing gastroesophageal varices, hepatic encephalopathy, and others. However, there is no consensus or literature describing how to manage asymptomatic patients with portosystemic shunts and normal liver.

**Case presentation:**

The patient was a 39-year-old female who underwent donor right hepatectomy for living donor liver transplantation. The patient was healthy by nature, however, developed hepatic encephalopathy after the surgery due to a development of portosystemic shunt. Portosystemic shunt stole portal blood flow, and imaging modalities revealed narrowing of the portal trunk, representing prolonged depletion of portal blood flow. Balloon-occluded retrograde transvenous obliteration (B-RTO) was performed for occlusion of the portosystemic shunt. B-RTO increased portal blood flow, and hepatic encephalopathy with hyperammonemia was successfully resolved without the outbreak of any other symptom of portal hypertension.

**Conclusions:**

A congenital portosystemic shunt itself is not a contraindication for donor hepatectomy, but perioperative endovascular shunts occlusion or intraoperative ligature of these shunts should be considered.

## Introduction

Living donor liver transplantation (LDLT) has become an established procedure for treating patients with end-stage liver disease; however, donor safety is a main concern. Careful consideration is necessary to avoid additional complications for otherwise healthy living donors.

Congenital portosystemic shunt (CPS) is an infrequent abnormal connection between the portal vascular system and the systemic circulation. Blood from the abdominal organs, which should drain via the portal vein into the liver is instead shunted to the systemic circulation by the portosystemic shunt. Complications of CPS include hepatic encephalopathy, pulmonary hypertension, liver tumors, and others. In Japan, neonatal screening for hereditary galactosemia is routinely performed, and portosystemic shunt occlusion for such cases with hepatic encephalopathy has been performed. The incidence of congenital portosystemic shunts has been estimated at 1 in 30,000 births [1]. Large cohort studies of CPS indicated that shunt closure should be considered for both therapeutic and prophylactic reasons [2]. However, there is no consensus or literature describing how to manage asymptomatic patients with a normal portal vein and a large congenital portosystemic shunt, such as a gastro-renal shunt. We report a case of prolonged depletion of portal blood flow and hepatic encephalopathy aggravated by a congenital portosystemic shunt after donor right hepatectomy in a patient who was successfully treated by balloon-occluded retrograde transvenous obliteration (B-RTO).

## Case presentation

**Fig. 1:**
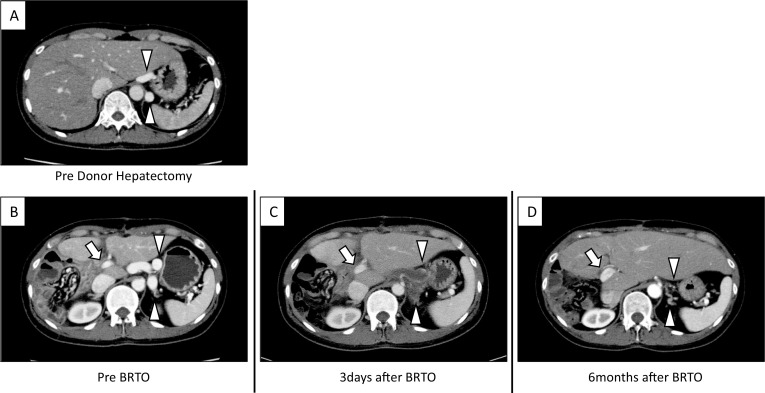
Contrast-enhanced computed tomography before and after B-RTO. **A** Preoperative contrast-enhanced computed tomography revealed a congenital portosystemic shunt 9 mm in diameter (arrowheads) originating from the left gastric vein (LGV) and draining to the left renal vein (LRV). **B**–**D** After donor hepatectomy, the portal trunk narrowed and the congenital portosystemic shunt widened 12 mm in diameter. After the B-RTO, the portal trunk expanded (arrows), and liver regeneration accelerated after balloon-occluded retrograde transvenous obliteration (B-RTO). Embolization of the LGV–LRV was successfully achieved and became inconspicuous 6 months after B-RTO (arrowheads)

**Fig. 2:**
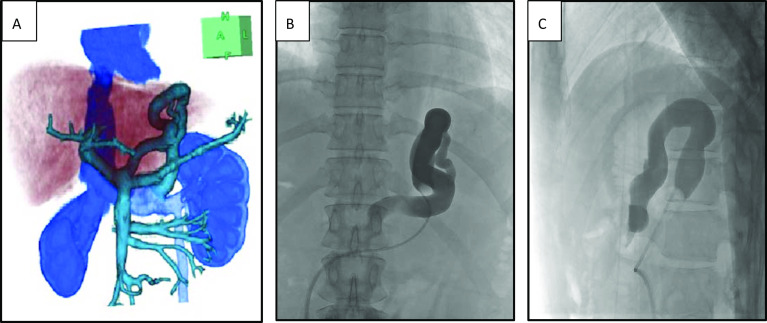
Postoperative MD-CT and fluoroscopic imaging during B-RTO. **A** Three-dimensional angiography by multidetector-row computed tomography (MD-CT) revealed a dilated congenital portosystemic shunt before balloon-occluded retrograde obliteration (B-RTO). **B**, **C** Fluoroscopy, coronal view (**B**) and sagittal view (**C**), confirmed that the balloon catheter was wedged in the portosystemic shunt, and the shunt was enhanced in a retrograde manner with 15 ml of 5% ethanolamine oleate iopamidol mixture

A 39-year-old female was a living donor for liver transplantation. The patient had no comorbidity and the volumetric analysis using preoperative multidetector-row computed tomography (MD-CT) showed that the remnant liver volume was 429 ml (40.5%) with normal structure of portal vein, and dilated congenital portosystemic shunt. The portosystemic shunt had a diameter of 9 cm, draining to the left renal vein (LRV) from left gastric vein (LGV) (Figs. 1A, 2A). The preoperative laboratory test recorded no abnormal results. The patient underwent donor right hepatectomy. The surgical procedures and techniques of living donor hemi-liver procurement via upper midline incision were described in our previous report [3]. Intraoperative findings revealed that the liver had normal appearance. The operative time was 320 min, and the estimated blood loss was 245 ml, the patient tolerated the procedure well and was sent to the surgical ward in stable condition.

Nine days after the surgery, the patient suffered from loss of motivation and memory, that was minimal encephalopathy (Grade 1; described by West Haven Criteria) and a high systemic blood ammonia level (maximum: 150 μg/dl). Postoperative MD-CT and three-dimensional angiography by MD-CT revealed development of portosystemic shunt (Fig. 1B). There was no obvious portal vein thrombosis; however, the portal trunk was narrowed and the portal blood flow was deteriorated (337 ml/min; measured with Doppler ultrasonography). It was suspected that portal flow stole to the congenital portosystemic shunt. The prothrombin time-international normalized ratio (PT-INR) was elevated to 1.24 INR, and D-dimer concentrations were also elevated to 17.5 μg/ml, suggesting a portal microthrombus caused by stagnant portal blood flow.

B-RTO was performed to obliterate the portosystemic shunt via the right femoral vein approach, as described previously [4]. A balloon catheter for B-RTO (6.5 French; Create Medic, Tokyo, Japan) was introduced via long sheath (8.0 French; Create Medic, Tokyo, Japan) into the LGV–LRV shunt. The LRV was the only draining route, so a balloon catheter wedged inside the shunt was enough to block the draining blood flow. Angiography using Iopamidol® visualized the congenital portosystemic shunt (Fig. 2B, C). Wedged pressure (portal pressure) was confirmed 13 mmHg, which was below 25 mmHg representing tolerable for shunt occlusion. We finally decided to occlude the LGV–LRV shunt. We injected 15 ml of 50% glucose and 2 ml of absolute ethanol, then 15 ml of 5% ethanolamine oleate iopamidol mixture was injected slowly in a retrograde manner. After overnight catheter placement, we deflated the balloon and confirmed the complete thrombosis of the shunt.

Postoperative course was uneventful, hepatic encephalopathy resolved completely, and the hyperammonemia improved the day after B-RTO. PT-INR also normalized promptly after the procedure. MD-CT on day 3 showed a good complete embolization of the LGV–LRV shunt (Fig. 1C). The patient was discharged on postoperative day 18, 9 days after the B-RTO, without any complications. Six months after B-RTO, MD-CT revealed an enlarged portal trunk and accelerated regeneration of the remnant liver, accompanied by the LGV–LRV shunt elimination (Fig. 1D). Upper gastrointestinal endoscopy and MD-CT revealed neither gastroesophageal varices nor dilation of other collateral vessels. These therapeutic effects lasted for more than 2 years of follow-up.

## Discussion

Portosystemic shunt, which is usually caused in cirrhotic patients by portal hypertension, is known as causing gastric varices or hepatic encephalopathy with high systemic blood ammonia. Congenital portosystemic shunt is rare, but also could be one of the causes of hepatic encephalopathy [5]. In the setting of living donor liver transplantation (LDLT), we obstruct the recipients’ large portosystemic shunt to ensure adequate graft inflow [6]. However, there is no consensus or literature describing how to manage asymptomatic patients with a normal portal vein and a large congenital portosystemic shunt in major liver hepatectomy.

Interventional radiology for portosystemic shunt, such as B-RTO has developed as a minimally invasive treatment for gastric varices [7]. And the indication of the procedure has spread to cure the hepatic encephalopathy caused of large portosystemic shunt [8, 9]. Recently, B-RTO might be feasible and recommended for both evaluation and treatment of congenital portosystemic shunt if possible [5], because the congenital portosystemic shunt might be the cause of hepatic encephalopathy, pulmonary hypertension, liver tumors, and others. However, there is no consensus or guideline describing how to manage asymptomatic patients with large congenital portosystemic shunt.

Various collateral pathways of portosystemic shunts contribute to the comorbidities, such as hepatic encephalopathy [10], and the drainage veins of portosystemic shunts mainly include: left renal vein, subphrenic vein, azygos vein, epigastric vein, direct shunt to the IVC, gonadal veins, iliac veins, among others. B-RTO is usually performed through the femoral vein. Transjugular venous approach is also reported to be useful for B-RTO [11]. An endovascular approach to manage portosystemic shunts is minimally invasive; however, other treatment modalities, such as surgical ligation, should be considered when the portosystemic shunt is not accessible. MD-CT is useful for characterizing the development of portosystemic shunts and determining the optimal method of treatment [12].

As for the embolus material, B-RTO has originally established as sclerotherapy using ethanolamine oleate with overnight catheter placement. Coil-assisted or plug-assisted retrograde transvenous obliteration has been also reported to avoid overnight catheter placement for patients with hepatic encephalopathy who could not keep complete bed rest. Overnight catheter placement might be painful for patient. However, we decided that it would not be acceptable to leave any artifacts in an otherwise healthy donor.

After the obliteration of portosystemic shunts by B-RTO, liver function, including hepatic encephalopathy, has been reported to be alleviated when the patients had enough hepatic reserve. Obliteration of portosystemic shunt by B-RTO accelerates portal blood flow and elevates the pressure [13–15]. Patients with enough hepatic reserve to buffer increased portal blood flow could recover their liver function [13, 14]. However, in patients with end-stage liver disease, this treatment could be not only less effective, but also deleterious [8]. Excessive portal blood flow followed by elevated portal vein pressure causes alternate collaterals such as gastroesophageal varices or ectopic varices [16]. These dilated collaterals increase the risk of bleeding, portal hypertensive gastropathy, and ascites. Before the occlusion, it must be evaluated to ensure that it does not result in excessive portal vein pressure. In this case, the patient was healthy by nature and considered to have enough hepatic reserve. The occlusion test confirmed that portal vein pressure, measured by the wedged pressure of portosystemic shunt, was less than 25 mmHg. Therefore, B-RTO was applied to treat hepatic encephalopathy as previously reported [17].

If the depletion of portal blood flow had prolonged and outbroke portal vein thrombosis, this patient’s condition became similar to patients with congenital extrahepatic portosystemic shunts [18]. Though the prognosis of patients with congenital extrahepatic portosystemic shunts is not so poor, hepatic encephalopathy, hepatopulmonary syndrome, and pulmonary arterial hypertension may develop, and some patients undergo as far as liver transplantation [2]. After the occlusion of portosystemic shunt, the hepatic encephalopathy resolved as expected. And the narrowed portal trunk expanded despite the development of PVT or deteriorate of liver function. Those results occurred as a result of increased portal blood flow. Furthermore, other no collateral vessels nor gastroesophageal varices developed after the procedure.

From this case and the experiences of the B-RTO, we propose the algorism of treating such donor candidates with congenital portosystemic shunt. First, the candidates with portosystemic shunt were contraindication if other candidates were acceptable, or the candidates suspected low liver reserve. The liver reserve could be estimated with not only the simple blood sampling tests but indocyanine green elimination test or ultrasound elastography. Needless to say, it is essential to evaluate for intrahepatic hemodynamic abnormalities. Second, donor candidates had enough liver reserve even with portosystemic shunt, and it is crucial to simulate preoperatively how to embolize the shunt when necessary. B-RTO might be a feasible strategy for perioperative shunt occlusion if the endovascular approach was possible. Otherwise, intraoperative surgical ligation of the portosystemic shunt might be necessary.

## Conclusions

We present a case for managing portosystemic shunt in a patient who underwent donor hepatectomy. A congenital portosystemic shunt itself is not a contraindication for donor hepatectomy, but precaution against congenital portosystemic shunt is necessary.

## Data Availability

Not applicable.

## Abbreviations

CPS: Congenital portosystemic shunt
B-RTO: Balloon-occluded retrograde transvenous obliteration
LDLT: Living donor liver transplantation
MD-CT: Multidetector-row computed tomography
LGV: Left gastric vein
LRV: Left renal vein
AST: Aspartate transaminase
ALT: Alanine transaminase
